# Supplementary figures and images for: MetaNovo: An open-source pipeline for probabilistic peptide discovery in complex metaproteomic datasets
Source: PLoS Comput Biol. 2023 Jun 16;19(6):e1011163. doi: 10.1371/journal.pcbi.1011163 (PMC10310047; doi:10.1371/journal.pcbi.1011163)

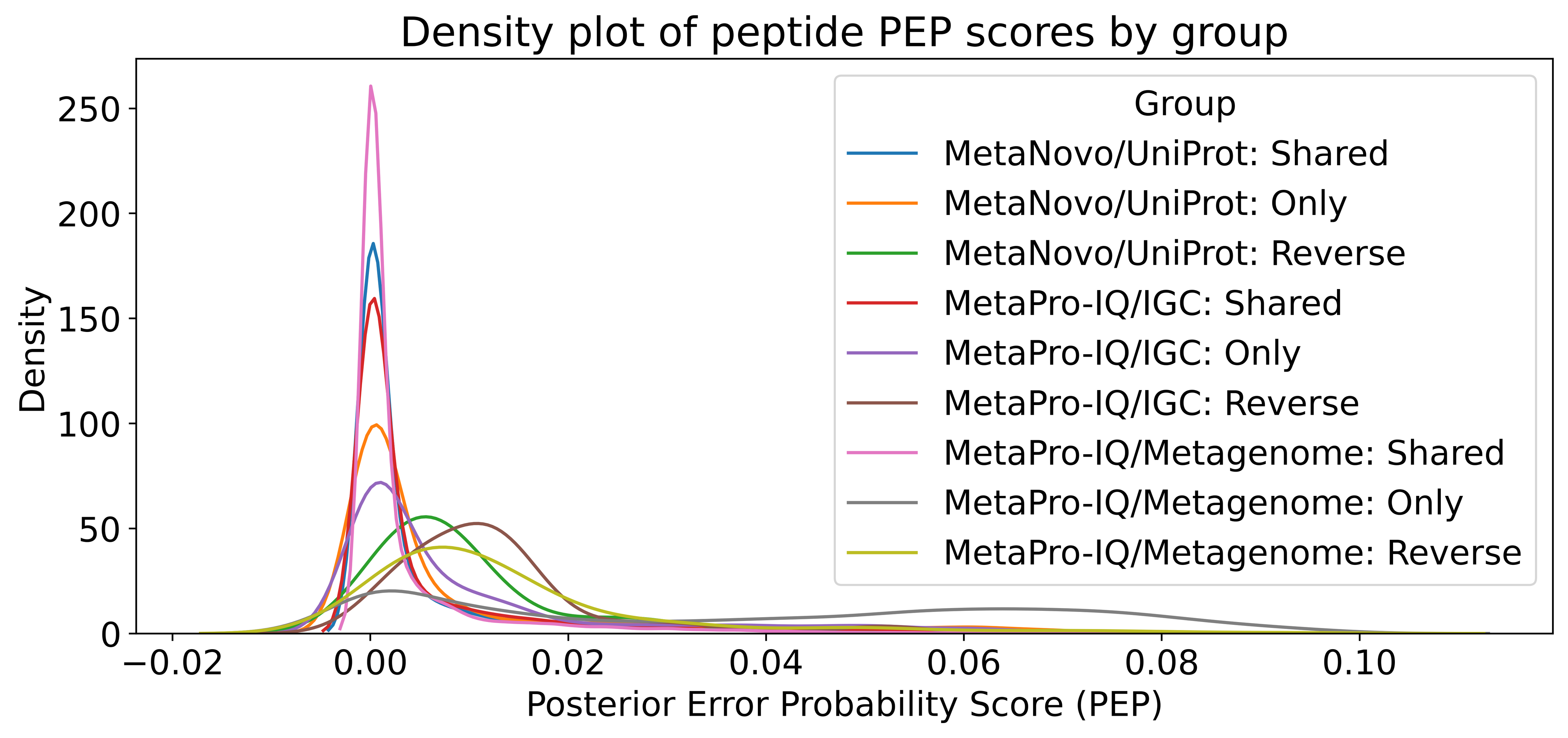

Supplement: S1 Fig — (TIF) [file pcbi.1011163.s015.tif]

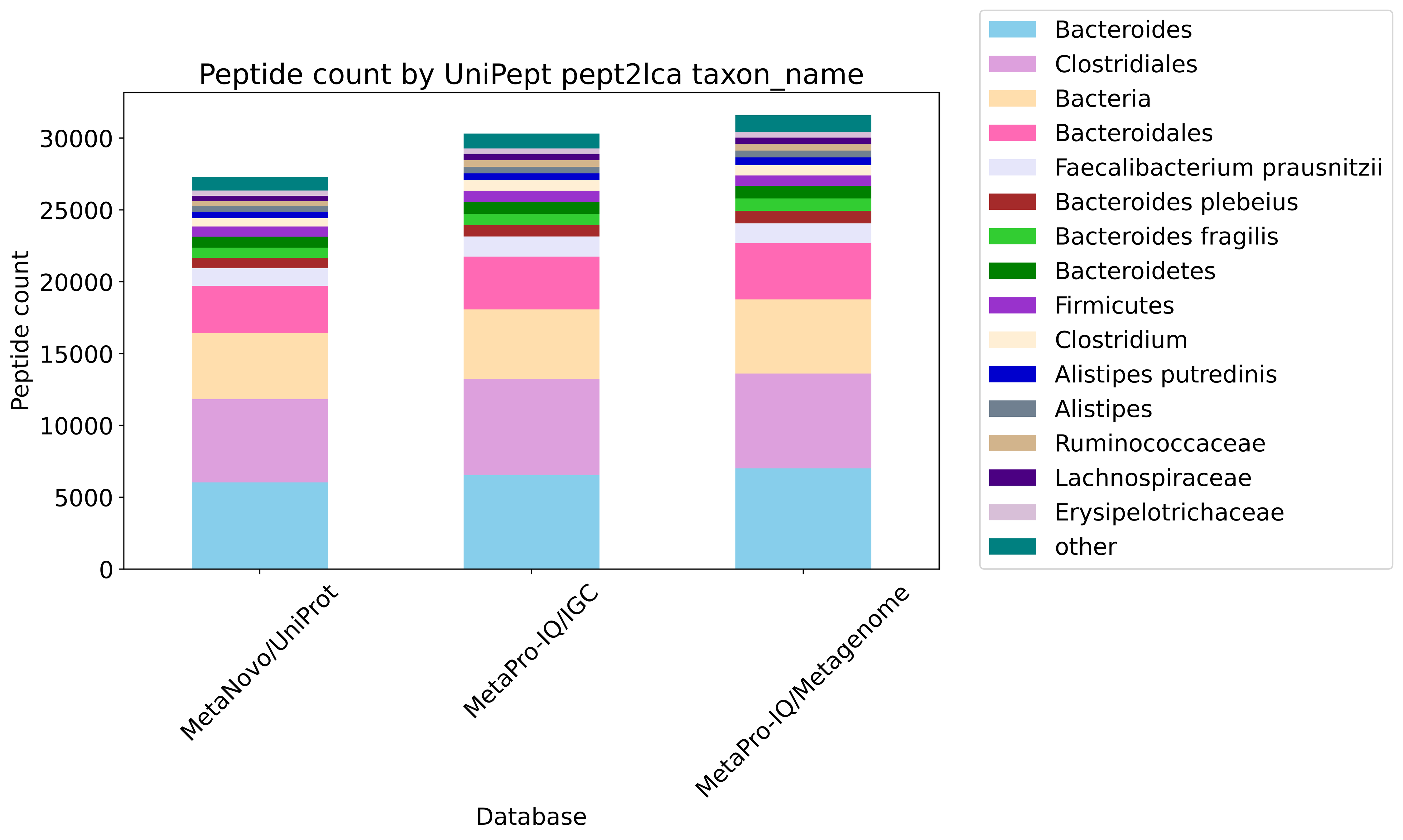

Supplement: S2 Fig — (TIF) [file pcbi.1011163.s016.tif]
