# Supplementary material for: MetaNovo: An open-source pipeline for probabilistic peptide discovery in complex metaproteomic datasets
Source: PLoS Comput Biol. 2023 Jun 16;19(6):e1011163. doi: 10.1371/journal.pcbi.1011163 (PMC10310047; doi:10.1371/journal.pcbi.1011163)
Supplement: Attachment — Submitted filename: Letter of Responses to Reviewers Comments .docx [file pcbi.1011163.s019.docx]

**Letter of responses to all review comments, and a description of the changes you have made in the manuscript.**

Reviewer's Responses to Questions

**Comments to the Authors:**

**Please note here if the review is uploaded as an attachment.**

Reviewer #1: *In this manuscript, Potgieter et al developed a metaproteomic data analysis tool, MetaNovo, which involves de novo sequencing in the first step for the refined database generation. MetaNovo enables peptide identification from a comprehensive Uniprot database directly. This method is widely applicable to a variety of multi-species samples. The peptides from the host and other contaminants can also be identified simultaneously, which can improve the identification rate and reduce the random matches. This work can help researchers in various fields aiming to study the proteome from multi-species samples. There are some minor concerns that need to be addressed before publication.*

**1. The users care about how fast the software runs. Could the authors provide the running time for the generation of the refined database from the Uniprot database by the de novo strategy?**

Thank you. We have updated the Manuscript *Design and Implementation* sections with running times.

The 9MM experiment was run on a single node of a high-performance computing cluster^[1]^, with 120 GB available RAM, and 8 allocated Intel Xeon Processor (Skylake, IBRS) processors. The total running time was 0d09h11m to process the 2 MGF files against the December 2019 release of UniProt.

The MetaProIQ experiment was run on a single node of a high-performance computing cluster^[2]^, with 120 GB available RAM, and 8 allocated Intel Xeon Processor (Skylake, IBRS) processors. The total running time was 3d03h38m to process the 8 MGF files against the December 2019 release of UniProt.

**2. Could the authors double-check the data used in Figure 3D and Figure 4D? It looks a little bit strange that there are parts of PSMs with higher PEP scores than the reverse parts. We know that the reverse parts represent the random matches, and if the “metaproiq-metagenome: only” in Figure 3D and the “Meta-PA: only” in Figure 4D are also random matches, the PEP distributions will be similar. However, these two parts showed significantly higher PEP scores than random matches. Besides, this trend was observed from two different datasets and was surprisingly consistent. If it’s correct, can you explore the reasons for this result?**

Thank you. The PEP score analysis was based on PEP scores from the MaxQuant *peptides.txt* column (as the *msms.txt* table was not available from the MetaProIQ PRIDE submission). As the PEP scores from the *peptides.txt* table are derived from the best-scoring MS/MS for each peptide sequence, and PSM level FDR was applied, it is possible for a group of peptides to have poorer median PEP scores compared to reverse hits at the peptide level. Even though the median PEP score of the 6525 target peptides only identified using the **MetaProIQ/Metagenome** run was worse than the 206 reverse hits of the same run, the difference was not statistically significant (Kruskal-Wallis and Dunn’s Test post hoc analysis p-value 0.46). A similar situation was found for the 9MM samples, where the median PEP score obtained from the **Meta-PA** only group (0.046675) was higher than the median peptide PEP score for reverse hits (0.010709), but the p-value for the difference in the distributions was not statistically significant (p=0.81). It is reassuring that in no comparison where the median PEP score was higher than for reverse hits, was the difference in the distribution statistically significant. It is interesting, in each case, that it was the database that yielded the poorest MS/MS identification rate, that yielded the poorest PEP scores for peptides exclusively identified using that database (not statistically significantly different from reverse hits) for both the **MetaProIQ/Metagenome**, as well as the **Meta-PA** 9MM database runs. We suggest that due to poor database representativity, PSMs to incorrect sequences in the database occur due to the non-inclusion of the correct sequences, with peptides exclusively identified by the incomplete databases thus having much poorer peptide PEP scores than the same groups obtained from the other, more complete databases, with peptides only identified by the most representative databases (by MS/MS percentage assignment) having the best median PEP scores in this category.

**3. Page 7, line 160. “for example using 16S sequencing data to produce a focused metaproteome library to search”. Here the word “library ” may cause a misunderstanding that it means a “spectra library”. I think it’s better to replace it with “database”.**

Thank you, we have made the change in the manuscript.

**4. Page 24, line 526-527. “The results were almost double those of the metagenome DB (Meta-PA) database that yielded 23.03 % identified MSMS, 6044 target peptides and 1087 target proteins”. What’s the size of the Meta-PA database? Is the size of Meta-PA too big which leads to lower identification rate? How many peptides can be identified if you use the MetaNovo workflow based on the Meta-PA database?**

The size of the Meta-PA database was relatively small, with 13270 non-redundant entries, much smaller than the SGA-PA with 27164 non-redundant entries and slightly larger than the **MetaNovo** database, which contained 13236 entries. As the results from Meta-PA were much poorer than for the SGA-PA database, it is unlikely that the reduced MS/MS assignment was due to the database size, but instead that the database was not representative enough, which would lead to poorer representativity with further database size reduction**.** This is backed up by the total lack of eukaryotic sequences in this database reported by the original authors, although the samples being analysed contained 2 yeast organisms, pointing to poor sequencing coverage of all organisms in the samples.

**5. Page 26, line 551. “or UniPept misassignments of natural variants in the data.” The database used here is a Uniprot database, which incorporates taxonomic information. Are the identified protein names the same as the UniPept results? Actually, considering the Uniprot database contains the information of the organism for each protein, why use UniPept in the workflow instead of using the taxonomic information directly?**

The UniPept *pept2lca* algorithm uses tryptic peptides, that may be specific to various taxonomic levels. Although certain peptides may belong to a UniProt protein annotated to a given species, those peptides may be shared with other proteins, belonging to other taxa, that may not have been included in the search database. Further, homologous proteins belonging to various taxa may share the same set of identified peptides in common, and using the *pept2lca* algorithm will allow the lowest common taxonomic level for each of those peptides to be assigned. We further chose the UniPept approach because it corresponds to the methodology used by the previous study on the 9MM samples.

**6. Page 33, line 714-716. The ethics statement mentioned that adult stool samples were used, but no related content was found in the article. Only two public datasets were downloaded and analyzed in the manuscript.**

Thank you, the adult stool samples referred to were from a previous iteration of the current manuscript and were since removed from the publication, in favour of publicly available datasets. We have removed the reference.

*Reviewer #2: I think this paper contributes an important methodology to the proteomics and metaproteomics fields of study. It proposes that instead of making assumptions about the composition of organisms in a metaproteomics sample, we can use the vast amount of sequenced proteins that are publicly available and let the mass spectrometry data speak for itself. They do this by using de novo sequencing of peptides, followed by tag matching to the massive database of UniProt, and then they use their scoring and ranking metric to determine the which proteins should be put in the targeted database. Next, they take advantage of the standard database search of the mass spectrometry data, followed by using UniPept to determine what organism were present in the sample.*

*While a 2% increase in the number of identified MS/MS spectra is not astonishing, the fact that this pipeline allows the user to not make any assumptions on the composition of their sample is immensely useful. This research is of high importance to many different areas of research where metagenomics and metaproteomics are used as tools to study complex biological systems. MetaNovo is particularly applicable if a lab does not have to budget or expertise to do metagenomics sequencing in addition to metaproteomics. The authors showed that they were able to detect chicken egg yoke proteins from the media that their sample was cultured on. This indicates that the MetaNovo method will also provide a better list of contaminate proteins.*

*I was able to access their GitHub code and their PRIDE data. While their code base is not thoroughly documented, it is well organized and readable, and their GitHub page has instructions for how to install and run the MetaNovo software. Between their supplemental material, raw data on PRIDE, and their public GitHub code I believe that this study is repeatable by the scientific community.*

*The authors come to reasonable conclusions that are backed up by their data, both in their figures and supplemental material. Just from my own curiosity, I’d be interested in seeing approximately how much time the MetaNovo pipeline takes to run on three replicate MGF files on a personal workstation or on a cluster.*

Dear reviewer, we have updated the manuscript and included the runtimes of the **MetaNovo** pipeline on the 2 9MM MGF files, as well as the 8 MLI samples, on a cluster. We hope this will give readers an accurate estimate of the software running time.

*Finally, line 760 says ‘S8_table.csv’ but the file downloaded is S8_figure.png.*

Thank you, we have renamed it to S8_figure.png

*Reviewer #3: This paper describes the algorithms, implementation, and results of a bioinformatics tool that uses de novo peptide identification to analyze LC-MS/MS metaproteomics data, specifically to create a database of protein sequences to use with traditional proteomics database search. The results show that the new tool has some advantages over more traditional methods of creating databases for metaproteomics/microbial community samples. The methods are sound and original, the results are interesting and the paper should be published. However, there are a few revisions that the authors could make to make their paper easier to understand. The authors should consider the following points:*

**1. Figures. Graphics resolution is low and type is very small in figures 3, 4, and 5. The small type is extremely hard to read and presents a real obstacle to understanding the paper. Authors should consider reformatting the figures, or breaking up multipart figures into multiple figures, to allow a larger font size. Possibly this is partly due to the automatic formatting in to a PDF file for reviewers, but as provided to the reviewer, the font size is too small. In addition, many plots are missing y-axis labels.**

Thank you, we have significantly increased the fonts for these figures. We have added y-labels as well.

**I also found Figure 2 to be difficult to understand. What does the arrow represent? Do the colors encode important information? Authors should consider revising the graphic, significantly expanding the caption, or possibly both. Also consider making the y-axis of Figure 3D and 4D logarithmic to should differences at lot PEP values better. Captions should include the dataset used.**

We have explained the arrow, and the meanings of the colours explicitly in the figure, and added some more explanation of the figure. We have made the y-axis of Figure 3D and 4D logarithmic. We have indicated the datasets in the captions.

**2. Dangers of omitting present organisms from the search database (page 8, lines 184-191). The authors cite Knudsen and Chalkley (reference 9 in the manuscript) as an example of what can happen when a true component of the sample is omitted from the search database. In this paper, Knudsen and Chalkley reexamine the data of Bromenshenk et al., who looked for protein markers of honeybee pathogens without including the honeybee protein database in their database. Knudsen and Chalkley do reach the conclusion that this omission was the cause of incorrect peptide identifications. However, Noble (**[**https://www.nature.com/articles/nmeth.3450**](https://urldefense.com/v3/__https://www.nature.com/articles/nmeth.3450__;!!JFdNOqOXpB6UZW0!uPZ6E51SXh-98-g8SDj4b6An5celcy8rDfyT4zaAyDitshS1qWM9FuZ36bQInzeF2bLRvCnwEODxilw_$)**) also reexamined the Bromenshenk data and reached a different conclusion: that only a small number of spectral assigned to pathogens should have been assigned to honeybee peptides, and “the choice of database does not explain the incorrect results obtained by Bromenshenk et al.” (Noble). Since the authors invoke Knudsen and Chalkley’s conclusion as a reason for the increased success of MetaNovo (page 28 line 603), I would be very interested to hear how they account for Noble’s arguments in this context.**

We agree in general with Bill Noble that the likelihood of misassigning an MS/MS spectrum truly derived from fragmentation of a eukaryotic (e.g. human or honeybee) peptide to a bacterial peptide is relatively low - even if that human peptide is absent from the search space - due to a general lack of sequence homology. However, an important distinction between the honeybee pathogen case re-evaluated by Noble and metaproteomic analyses is that in the latter, the absence of a ‘true hit’ bacterial peptide from the search space is much more likely to lead to false positive IDs due to significant homology with orthologous proteins in the bacterial kingdom which are in the search space but that are not truly in the sample, yet such false positives would be expected to have lower PEP scores. Furthermore, ‘true hit’ peptides (bacterial or human) that are absent from the search space can by definition not be found through peptide-spectrum matching, resulting in false negatives. Thus, **MetaNovo** solves both false positive and false negative ID problems by providing the means to search a vast, more complete sequence space without compromising FDR estimation. These possibilities were not considered in the Noble analysis so there is in fact no contradiction between our conclusions and Noble’s arguments; we have included a discussion on this in the revised manuscript.

**3. What do you mean by database? When describing each data set analyzed, the authors indicate the size of the search database produced by MetaNovo (e.g. page 17 lines 374-376 for the mucosal-luminal interface dataset). If I am following correctly, this “database” is the product of the whole MetaNovo pipeline, the “parsimonious protein subset identified” indicated near the bottom center of Figure 1. There is some possibility for the reader to confuse this database with the SQLite database that holds all the the DirecTag results, described on page 13 lines 287-291. The authors should consider how to distinguish these two uses of the word “database” to ensure that the results section is clear. I’m not just trying to nitpick here, it actually confused me the first time through.**

We have explicitly replaced the use of “database” with either “sequence database” or “SQLite database” in the text, where appropriate to clarify the meaning.

**4. Page 23 line 501. Clarify what is meant by “taxon_name taxa.”**

We have defined this term in the text. UniPept *pept2lca taxon_name* is the most specific possible taxonomic characterization for a given peptide across all taxonomic levels identified by the algorithm.

1. https://www.ilifu.ac.za/
2. https://www.ilifu.ac.za/
